# Botulinum toxin injection for the treatment of chronic anal fissure: uni- and multivariate analysis of the factors that promote healing

**DOI:** 10.1007/s00384-022-04110-0

**Published:** 2022-02-11

**Authors:** Giuseppe Brisinda, Maria Michela Chiarello, Anna Crocco, Anna Rita Bentivoglio, Maria Cariati, Serafino Vanella

**Affiliations:** 1 Università Cattolica del Sacro Cuore, Roma, Italy; 2 Dipartimento Di Scienze Mediche E Chirurgiche, Fondazione Policlinico Universitario A Gemelli, IRCCS, Roma, Italy; 3 Unità Operativa Di Chirurgia Generale, Ospedale San Giovanni Di Dio, Crotone, Italy; 4 Unità Operativa Di Chirurgia Oncologica Della Tiroide E Della Paratiroide, Istituto Nazionale Tumori, IRCCS Fondazione Pascale, Napoli, Italy; 5 Unità Operativa Di Neurologia, Fondazione Policlinico Universitario A Gemelli, IRCCS, Roma, Italy; 6 Unità Operativa Di Chirurgia Generale E Oncologica, Azienda Ospedaliera San Giuseppe Moscati, Avellino, Italy

**Keywords:** Chronic anal fissure, Botulinum toxin, Lateral internal sphincterotomy

## Abstract

**Purpose:**

Anal fissure is caused by a pathological contraction of the internal anal sphincter. Lateral internal sphincterotomy remains the gold standard for the treatment of fissure. Botulinum toxin injections have been proposed to treat this condition without any risk of permanent injury of the internal sphincter. We investigate clinical and pathological variables and the effects of different dosage regimens of botulinum toxin to induce healing in patients with idiopathic anal fissure.

**Methods:**

This is a retrospective study at a single center. The patients underwent a pre-treatment evaluation that included clinical inspection of the fissure and anorectal manometry. We collected and analyzed demographic data, pathological variables, associated pathological conditions, and treatment variables. Success was defined as healing of the fissure, and improvement of symptoms was defined as asymptomatic persistent fissure.

**Results:**

The findings of 1003 patients treated with botulinum toxin injections were reported. At 2 months evaluation, complete healing was evident in 780 patients (77.7%). Resting anal tone (77.1 ± 18.9 mmHg) was significantly lower from baseline (*P* < 0.0001) and from 1-month value (*P* = 0.0008). Thirty-nine not healed patients underwent lateral internal sphincterotomy, and 184 were re-treated with 50 UI of botulinum toxin. In these patients, the healing rate was 93.9% (171 patients). Dose and injection site of toxin correlates with healing rate. There were no relapses during an average of about 71 months.

**Conclusion:**

Our data show that injection of botulinum toxin into the internal anal sphincter is a safe and effective alternative to surgery in patients with chronic anal fissure.

## Introduction

Chronic anal fissure (AF) is an ulcer of the distal anal canal. Fissure causes substantial morbidity in otherwise healthy people [1]. Most of the fissures are located at the posterior commissure [2, 3]. Chronic AF is characterized by a marked reluctance to healing in the absence of treatment [4]. Spasm of the internal anal sphincter has been associated to chronic AF [5–7]. Treatment has focused on alleviating hypertonia of the sphincter. The most common surgical treatment is partial internal lateral sphincterotomy [8, 9]. A recent systematic review demonstrates that healing rates were 95.13% in patients treated with sphincterotomy, although the benefit of surgical procedure was at a cost of increased complications, notably permanent incontinence [2]. A drawback of this surgery is its potential to cause minor but sometimes permanent alterations in gas, mucus, and occasionally, stool control [10, 11]: Mechanisms of continence can be acutely decompensated by the sphincterotomy, and incontinence can affect up to 35% of patients in the postoperative period [12–17].

To avoid the permanent sequelae of sphincterotomy, alternative treatments can be adopted—such as the injection of botulinum toxin or the topical application of nitro derivatives—to induce the healing of the fissure without permanent damage to the anal sphincters [18–23]. Several studies have documented that botulinum toxin injection is effective in the treatment of chronic AF [24–27]. Furthermore, a recent survey by the American Society of Colon and Rectal Surgeons have been documented that the injection of botulinum toxin for chronic AF is commonly used by colorectal surgeons and that there is a consensus on toxin dosage, administration site, technique, and also on the use of monitored anesthesia care [21].

We report the results of a retrospective non-randomized study of patients undergoing botulinum toxin injections for the treatment of chronic AF, in a 10-year period. We collected and analyzed demographic data, pathological variables, associated pathological conditions, and treatment variables. To identify variables that promote the healing of AF, a univariate and multivariate analysis was performed.

## Methods

This is a retrospective study in a single center. A total of 1003 consecutive patients with symptomatic chronic AF were enrolled in the study. The diagnosis was based on the following clinical criteria: evidence of posterior circumscribed ulcer with a hypertrophic sentinel tag of skin, exposure of the horizontal fibers of the internal anal sphincter, and typical symptoms (post-defecatory pain, pain at a distance from evacuation and/or at night, bleeding) lasting for more than 2 months. Each patient had provided written informed consent.

Patients with acute fissure or chronic AF associated with other conditions (i.e., chronic inflammatory bowel disease, HIV infection, tuberculosis, sexually transmitted diseases, immunosuppression, and/or anorectal or perianal cancer) were excluded. Other exclusion criteria were systemic diseases with altered cholinergic transmission, use of medication capable of interfering with the action of the toxin (aminoglycosides, baclofen, dantrolene, or diazepam), pregnancy and/or breast-feeding, or known hypersensitivity to the components of the formulations.

### Clinical evaluation and baseline assessment

Every patient underwent a pre-treatment evaluation that included clinical inspection of the fissure and anorectal manometry. Demographic data on age and gender were recorded in all patients. Data regarding anal pathology (duration and characteristics of the symptoms), association with other anal and/or systemic pathologies, allergies, and previous interventions were recorded for each patient. Also, stool consistency, the frequency and peculiarity of evacuations, and the use of laxatives or enemas were recorded for each patient. The resting and maximal squeeze anal canal pressures (i.e., the maximal voluntary increase in pressure above the resting pressure) were measured by stationary pull-through techniques.

### Preparation of the toxin and method of injection

The botulinum toxin type A was diluted with saline solution. When the American formulation (Botox®; Allergan, Irvine, California, USA) was used, the vials contained 100 IU of type A botulinum toxin stored at a temperature of -20 °C and diluted with saline solution at 50 IU/ml, at the time of use; when the European formulation (Dysport®; Spywood Biopharm, Wrexham, UK) was used, the vials contained 500 freeze-dried IU of type A botulinum toxin diluted in saline solution at 150 IU/ml. A conversion factor of 1 to 3 (1 IU Botox = 3 IU Dysport) was adopted [28]. This conversion factor is due to the different potency of the two toxin preparations. In the text, when not otherwise specified, reference is made to Botox UI. After manually detecting the internal anal sphincter, the toxin was injected through a 27-gauge needle with the patient lying on his left side. The toxin was injected in the anterior or posterior commissure, or in both. No systemic sedation was administered during the procedure.

### Aim of the study

The primary outcome was the complete healing after treatment. The treatment was considered successful if the fissure healed. Persistence of the fissure in the absence of specific symptoms was considered a symptomatic improvement.

### Follow-up

Patients were re-examined at 1 and 2 months after the treatment. Each patient underwent a clinical evaluation and anorectal manometry. This approach was also used in patients who underwent rescue treatment. Clinical control was carried out by telephone interviews (once a year). All patients were given a dedicated telephone number to refer to in the event of specific symptoms (appearance of post-defecatory pain, bleeding after evacuation). Clinical visit was performed only in patients who complained of specific symptoms. In consideration of the reversibility of the treatment and, therefore, the reversibility of any complication, no score for the evaluation of fecal continence was used.

### Statistical analysis

Data were analyzed using standard statistical methods. Initially, a univariate analysis of all potential factors influencing the course of the disease was performed using the Chi-square test or Fisher’s exact test for categorical data and the ANOVA test for continuous data in groups of more than two. Prespecified subgroups in these analyses were defined according to age (≤ 35 years, 36 to 45 years, 46 to 60 years, or > 60 years), duration of symptoms before treatment (≤ 7 months or > 7 months), number of evacuation for week (≤ 7 or > 7 evacuations/week), site of injection of the toxin (anterior injection, posterior injection, combined setting), number of units injected (from 15 to 100 IU), and dosage groups (low ≤ 25 IU injected, medium 30 IU, and high 50–100 IU). Subsequently, a multivariate logistic regression was performed by building models that consider the potential factors influencing the healing rate that at univariate analysis had a value of *P* < 0.25, according to the Hosmer–Lemeshow rule. Data were processed using GraphPad® Prism software (GraphPad, San Diego, USA). A value of *P* < 0.05 was considered statistically significant, regardless of the test used.

## Results

Demographic data and clinical characteristics of 1,003 patients with chronic AF treated at the General Surgery Operative Unit, Fondazione Policlinico Universitario “A Gemelli” IRCCS of Rome are reported in Table 1. Regarding the characteristics of the bowel movements, 459 patients (45.8%) reported constipation, and 490 (48.9%) increased bowel movements. Number of evacuations per week was less than 7 in 88.3% of cases. A forced evacuation was reported by 489 patients (48.8%) and an incomplete evacuation by 262 patients (26.1%). At baseline, a resting anal tone of 102.4 ± 20.1 mmHg and a voluntary contraction of 85.4 ± 35.7 mmHg has been documented.

**Table 1:** Analysis of parameters in 1,003 patients undergoing treatment with botulinum type A toxin for chronic fissure. The frequency and percentage for each individual variable considered are shown

**Parameters considered**	**Frequency**	**%**	**Cumulative %**
**Age (years)** ≤ 35 36–45 46–60 > 60	285 247 293 178	28.4 24.6 29.2 17.7	28.4 53.0 82.3 100.0
**Sex** Male Female	574 429	57.2 42.8	57.2 100.0
**Duration of symptoms** ≤ 7 months > 7 months	618 385	61.6 38.4	61.6 100.0
**Post-operative pain** Present Absent	985 18	98.2 1.8	98.2 100.0
**Night-time pain** Present Absent	179 824	17.8 82.2	17.8 100.0
**Pain in the time** Present Absent	269 734	26.8 73.2	26.8 100.0
**Bleeding** Present Absent	678 325	67.6 32.4	67.6 100.0
**Loss of mucus** Present Absent	28 975	2.8 97.2	2.8 100.0
**Other anal disease** Present Absent	126 877	12.6 87.4	12.6 100.0
**Extra-anal disease** Present Absent	86 917	8.6 91.4	8.6 100.0
**Allergies** Present Absent	9 994	0.9 99.1	0.9 100.0
**Previous surgery** Yes No	204 799	20.3 79.7	20.3 100.0
**Bowel functions** Normal Diarrhea Constipation	497 47 459	49.6 4,7 45.8	49.6 54.2 100.0
**Consistence of stool** Shaped Soft Hard	472 41 490	47.1 4.1 48.9	47.1 51.2 100.0
**Number of weekly evacuation** ≤ 7 > 7	887 116	88.4 11.6	88.4 100.0
**Forced evacuation** Present Absent	490 513	48.9 51.1	48.9 100.0
**Incomplete evacuation** Yes No	263 740	26.2 73.8	26.2 100.0
**Use of laxative** Yes No	379 624	37.8 62.2	37.8 100.0
**Use of enemas** Yes No	235 768	23.4 76.6	23.4 100.0

In all patients, toxin was injected into the internal anal sphincter: dose and site of injection were reported in Table 2. In 94.1% of patients, no local anesthesia and/or sedation was required, and in 93.9% of patients, the sphincter was easily identified by simple digital palpation. Patients (5.9%) received subcutaneous administration of local anesthetic, prior to the toxin injection. In one case, a local reaction occurred, consisting of a limited and modest skin rash, which resolved after 4 days following topical application of corticosteroids.

**Table 2:** Results of treatment with botulinum toxin. Frequency and percentage are shown for each parameter

	**Frequency**	**%**	**Cumulative %**
**Dose of toxin (UI)** 15 20 25 30 50 100	89 112 3 196 599 4	8.9 11.2 0.3 19.5 59.7 0.4	8.9 20.1 20.4 39.9 99.6 100.0
**Dosing group** Low (15–25 UI) Medium (30 UI) High (50–100 UI)	204 196 603	20.4 19.5 60.1	20.4 39.9 100.0
**Site of injection** Posterior site Anterior site Combined site (posterior + anterior)	167 814 22	16.7 81.2 2.2	16.7 97.8 100.0
**Local reactions** Yes No	1 1002	0.1 99.9	0.1 100.0
**Local anesthesia** Yes No	59 944	5.9 94.1	5.9 100.0

One month after treatment, healing was observed in 751 patients (74.9%). A reduction in resting anal tone (80.3 ± 18.1 mmHg, *P* < 0.0001 vs baseline value) and maximal voluntary contraction (76.5 ± 23.9 mmHg, *P* < 0.0001 vs baseline value) was observed.

At the 2 months evaluation, complete healing was apparent in 780 patients (77.7%). Incontinence was not observed in any patient. Resting anal tone (77.1 ± 18.9 mmHg) was significantly lower from baseline (*P* < 0.0001) and from 1-month value (*P* = 0.0008). Maximal voluntary contraction (77 ± 26.4 mmHg) was lower than the baseline value (*P* < 0.0001) and did not vary from 1-month value (*P* = 0.6).

At 2-months evaluation, 223 patients (22.3%) were not healed. Of these, 39 patients underwent lateral internal sphincterotomy, and 184 were treated with further injection of 50 UI of botulinum toxin. In these latter group, the healing rate was 93.9% (171 patients). Incontinence has not been observed in any patient. Before the rescue treatment, a resting anal tone of 100.6 ± 27.2 mmHg, and a voluntary contraction of 84.5 ± 31.2 mmHg has been documented in all the injected patients. At 1-month evaluation after rescue treatment, resting anal pressure and voluntary contraction were 69.5 ± 17.6 mmHg and 83.3 ± 39.9 mmHg, respectively. At 2 months evaluation, resting anal pressure and maximal voluntary contraction were 68.5 ± 14.3 mmHg and 82.7 ± 38.6 mmHg, respectively. Resting anal pressure was significantly lower than the baseline values (*P* < 0.0001) and did not vary from 1-month value (*P* = 0.7). Maximal voluntary contraction did not differ significantly from baseline (*P* = 0.7) and from 1-month (*P* = 0.8) values.

The follow-up was performed as described above. Complete information for the first 3 years after treatment was obtained from 950 patients. None of them reported the appearance of symptoms and/or fecal incontinence, referred as uncontrolled losses of gas, mucus, and/or liquid stools. In the following years, 398 patients were lost to follow-up. The remaining 552 patients reported no post-defecatory pain or bleeding. All reported the absence of disorders related to fecal continence. The mean follow-up period was 71.4 ± 38.6 months.

At univariate analysis, no demographic parameters were shown to have a direct influence on healing (Table 3). Both age (*P* = 0.8) and sex (*P* = 0.5) showed no statistically significant difference. Among clinical variables, only pain after evacuation showed influence on the healing (*P* = 0.04). The dose and injection site of the toxin correlate significantly with the healing rate. Healing was not observed in any cases treated with 15 IU, in 71 out of 112 treated with 20 IU, in 2 out of 3 with 25 IU, in 159 out of 195 with 30 IU, and in 450 out of 563 treated with 50 IU (*P* < 0.0001). The subdivision of the patient population into the 3 dosage groups (high, medium, and low) documents a healing rate of 81.5% in medium dosage group, compared to 79.5% and 51% in those treated with high or low dosages, respectively (*P* < 0.0001). Another variable found to be strongly correlated with healing is the injection site: healing was shown in 56.6% of patients treated with posterior injections and in 78.6% of those with anterior injection of the toxin (*P* < 0.0001). Two months after treatment (Table 4), a statistically significant influence in healing was found in patients with persistence of pain after the evacuation (*P* = 0.02). Similarly, to 1-month evaluation, the dose significantly correlates with healing, observed in 29.2%, 75%, 79.3%, and 83.9% of patients treated with 15 IU, 20 IU, 30 IU, and 50 IU, respectively (*P* < 0.0001). In contrast to the 1-month evaluation, the highest dose group showed a higher healing rate (83.1%) than patients in the medium (79.3%) and low (64.8%) dose groups (*P* < 0.0001). At the 2-month evaluation, the injection site correlates statistically with healing (healing rate 59.2% and 82.1% in the posterior or anterior injection, respectively, *P* < 0.0001).

**Table 3:** Results of the univariate analysis after 1 month follow-up from the treatment with botulinum toxin

**Variable**	**Univariate analysis (*P*** **value)**
**Age**	0.874
**Sex**	0.524
**Duration of symptoms**	0.413
**Post-defecatory pain**	0.301
**Night-time pain**	0.570
**Pain at distance from the evacuation**	0.004
**Bleeding**	0.065
**Loss of mucus**	0.154
**Other anal pathology**	0.966
**Extra-anal pathology**	0.749
**Allergies**	0.635
**Previous surgery**	0.910
**Bowel function**	0.712
**Consistency of stools**	0.656
**Weekly evacuations**	0.131
**Forced evacuations**	0.311
**Incomplete evacuations**	0.377
**Laxative use**	0.282
**Use of enemas**	0.675
**Toxin dose**	< 0.0001
**Dosing group**	< 0.0001
**Site of injection**	< 0.0001
**Local reactions**	0.755

**Table 4:** Findings of the univariate analysis to 2 months follow up after treatment with botulinum toxin

**Variable**	**Univariate analysis (*P*** **value)**
**Age**	0.270
**Sex**	0.953
**Duration of symptoms**	0.566
**Post-defecatory pain**	0.257
**Night-time pain**	0.873
**Pain at a distance from the evacuation**	0.02
**Bleeding**	0.711
**Loss of mucous**	0.494
**Other anal disease**	0.888
**Extra-anal disease**	0.824
**Allergies**	0.189
**Previous surgery**	0.095
**Bowel function**	0.740
**Consistency of stools**	0.781
**Weekly evacuations**	0.272
**Forced evacuations**	0.311
**Incomplete evacuations**	0.192
**Laxative use**	0.228
**Use of enemas**	0.510
**Toxin dose**	< 0.0001
**Dosing group**	< 0.0001
**Site of injection**	< 0.0001
**Local reactions**	0.784

In the multivariate analysis (Table 5), the following variables were independent factors for healing rate: pain after evacuation (*P* = 0.0008) and dose of toxin injected (*P* < 0.00001). At 2 months, both the dose of toxin injected (*P* = 0.004) and the injection site (*P* = 0.01) correlate with the healing rate (Table 6).

**Table 5:** Results of multivariate analysis 1 month follow up from botulinum toxin treatment

**Variable**	**Multivariate analysis** **(*P*** **value)**	**T Ratio**	**95%CI**
**Age**	0.7664	0.2972	-0.002253 to 0.001660
**Sex**	0.2318	1.197	-0.02201 to 0.09102
**Pain at distance**	0.0008	3.361	0.04576 to 0.1738
**Bleeding**	0.0621	1.868	-0.002802 to 0.1166
**Loss of mucus**	0.2642	1.117	-0.07641 to 0.2790
**Evacuation/weekly**	0.8641	0.1712	-0.007941 to 0.009462
**Dose of toxin**	< 0.0001	4.816	0.003161 to 0.007500
**Site of injection**	0.1404	1.476	-0.1621 to 0.1150

**Table 6:** Results of multivariate analysis 2 months follow-up after treatment with botulinum toxin

** Variable**	**Multivariate analysis** **(*P*** **value)**	**T ratio**	**95%CI**
**Age**	0.3492	0.9369	-0.003446 to 0.001217
**Sex**	0.6985	0.3875	-0.05314 to 0.07933
**Pain at a distance from the evacuation**	0.0521	1.946	0.0005432 to 0.1502
**Incomplete evacuation**	0.1011	1.642	-0.01429 to 0.1619
**Laxative use**	0.0964	1.665	-0.1438 to 0.01169
**Previous surgery**	0.0465	1.995	-0.001418 to 0.1601
**Allergies**	0.6463	0.4591	-0.2508 to 0.4042
**Dose of toxin**	0.0046	2.842	0.001093 to 0.005948
**Site of injection**	0.0189	2.354	0.01485 – 0.1625

## Discussion and conclusions

Data confirmed that chronic AF affects otherwise healthy subjects [29]. The clinical picture is so characteristic that it is diagnostic. In our study, 985 patients complained of post-defecatory pain; bleeding was present in 676 patients (67.4%). Regarding bowel movements, 459 patients (45.8%) reported constipation and 490 (48.9%) evacuation of hard stools. In accord with literature [12], no marked incidence in one sex over the other or in different age groups have been observed.

Spasm of the internal anal sphincter has been observed in association with chronic AF. For many years, the aim of the treatment has been to reduce hypertonia of the sphincter. Botulinum toxin has been used to treat chronic AF while averting the risk of permanent injury of the internal anal sphincter [30–32]. The toxin induces a significant reduction in the resting anal tone [26, 32]. Furthermore, the toxin was shown to be superior to nitro derivatives in inducing healing of chronic AF [26, 27, 33, 34].

Botulinum toxin injection is easier to perform than surgery. In this series, in 94.1% of the treatments, no local anesthesia and/or systemic sedation was used, and in 93.9% of patients, the sphincter was easily identified with simple digital palpation. In 0.1% of cases, a local reaction occurred, consisting of a limited and modest skin rash, which resolved after 4 days following the topical application of corticosteroids.

The toxin can be used safely for the treatment of chronic AF, particularly in patients at risk of incontinence [24]. The toxin is a safe treatment and has several advantages over other methods [29]. In the present study, healing was observed in 74.9% of patients at 1-month evaluation. After 2 months, a complete healing was evident in 77.7% of patients. Healing remained stable for a period of 71.4 ± 38.6 months. No demographic parameters were shown to have a direct influence on healing. We did not observe AF recurrence in the patient population treated. This finding is probably linked to the fact that in most of our patients, the fissure had appeared less than 7 months before and that most of the patients had undergone treatment with at least 30–100 IU of toxin. Few studies assess long-term recurrence. Some of these studies document a trend to progressive recurrence over time, with lower healing rates than those initially reported [35]. These results could be related to the reversible effect of the toxin and the natural history of the disease. It has also been showed that recurrence occurred mainly between 6 and 12 months, so later relapses were not expected. Other findings in the literature proved that the efficacy of botulinum toxin was maintained during a long-term follow-up (5 years), with a negligible recurrence rate at 1 year (11.4%), and no recurrence at 3 and 5 years of follow-up [1].

The result of the present study also confirms that the use of higher doses and the availability of a rescue treatment account for a higher success rate, without any increase of complications or side effects. As previously reported, manometric data have shown that the internal anal sphincter is weakened after the injection and that no significant diffusion to the external sphincter takes place.

We observed that toxin dose significantly correlates with healing rate, as reported in other studies [36, 37]. The higher the dose used, at least up to 50 IU, the more conspicuous the number of patients healed. It has been documented that higher dosages of toxin induce healing in a larger number of patients, also determining a more significant decrease in resting anal tone. The reduction of the resting anal tone is, moreover, an event directly correlated to the toxin dose: The higher the dose used, the faster and more marked the decrease in resting anal tone. Diffusion of toxin in the tissue is a dose-dependent phenomenon. Histochemical staining of acetylcholinesterase suggested that higher doses produced a biological effect throughout the entire muscle, whereas smaller doses produced a gradient down the length of the muscle studied [38]. The dose of toxin injected directly correlates with the results [36, 37, 39, 40]. At univariate analysis, our study confirmed that the dose of toxin correlated significantly with healing. At 2 months evaluation, the higher dose group (50–100 IU) showed a higher healing rate (83.1%) than patients in the medium (79.3%) and low (64.8%) groups dose (*P* < 0.0001).

Another parameter strongly correlated with healing is the injection site. Healing was apparent in 56.6% of patients injected at the posterior midline and in 78.6% of those with toxin administration at the level of the anterior commissure, as reported in another study [41]. We hypothesized that botulinum toxin action on the internal anal sphincter depends upon the muscle fiber response and that the toxin is more effective in a non-ischemic sphincter. Brown and coworkers [42] have underlined that a myositis involving internal anal sphincter close to the fissure site can develop early in chronic AF patients, causing fibrosis. The fibrosis was more prominent at the base of the fissure; it could reduce compliance of the internal anal sphincter and might block the toxin action. Moreover the blood supply of the anoderm at the posterior midline was found to be significantly lower than the other sides of the anal canal. A significant relationship between anal pressure and anodermal blood flow was demonstrated at the posterior midline, and morphometric study of the capillaries revealed them to be less dense both in the subanodermal space and within the internal anal sphincter [43]. The myenteric plexus with myenteric ganglia was located between the circular and longitudinal smooth muscle layers for the whole extent of the internal anal sphincter. It seems conceivable that fibrosis or ischemia at the base of the posterior fissure may destroy the myenteric nervous fibers and, when using botulinum toxin in this site, a variable clinical response can be observed. Botulinum toxin may induce healing by simple increasing local blood flow or by a more complex effect. It has been shown that botulinum toxin does not block non-adrenergic noncholinergic responses, which are mediated by nitric oxide [30]. At multivariate analysis, a significant correlation was documented with the injection site (*P* = 0.01).

In this study, we did not observe any differences in the clinical efficacy of the two different preparations of botulinum toxin type A available on the market, at a conversion factor of 1 to 3 as reported in the literature for the treatment of affections of the striated muscles. These findings confirm our previous observation on 100 patients with chronic anal fissure [28].

In conclusion, we confirm that the botulinum toxin is a safe treatment for AF patients. We find it effective and easier to perform compared to surgery. The best results were obtained when, in patients with posteriorly located fissure, the toxin was injected into the internal sphincter at the anterior commissure. The optimal dose was found to be 50 IU of American toxin or 150 IU of British formulation. We have noted that higher dosages induce a statistically significant increase in the healing rate without increasing complications.
